# Prebiotic Potential of a Maize-Based Soluble Fibre and Impact of Dose on the Human Gut Microbiota

**DOI:** 10.1371/journal.pone.0144457

**Published:** 2016-01-05

**Authors:** Adele Costabile, Eddie R. Deaville, Agustin Martin Morales, Glenn R. Gibson

**Affiliations:** 1 Department of Food and Nutritional Sciences, University of Reading, Whiteknights, PO Box 226, Reading, RG6 6AP, United Kingdom; 2 Health Sciences Research Centre, Life Sciences Department, Whitelands College, University of Roehampton, London, Holybourne Ave, London, SW15 4JD, United Kingdom; University of Ulm, GERMANY

## Abstract

Dietary management of the human gut microbiota towards a more beneficial composition is one approach that may improve host health. To date, a large number of human intervention studies have demonstrated that dietary consumption of certain food products can result in significant changes in the composition of the gut microbiota i.e. the prebiotic concept. Thus the prebiotic effect is now established as a dietary approach to increase beneficial gut bacteria and it has been associated with modulation of health biomarkers and modulation of the immune system. Promitor™ Soluble Corn Fibre (SCF) is a well-known maize-derived source of dietary fibre with potential selective fermentation properties. Our aim was to determine the optimum prebiotic dose of tolerance, desired changes to microbiota and fermentation of SCF in healthy adult subjects. A double-blind, randomised, parallel study was completed where volunteers (n = 8/treatment group) consumed 8, 14 or 21 g from SCF (6, 12 and 18 g/fibre delivered respectively) over 14-d. Over the range of doses studied, SCF was well tolerated Numbers of bifidobacteria were significantly higher for the 6 g/fibre/day compared to 12g and 18g/fibre delivered/day (mean 9.25 and 9.73 Log10 cells/g fresh faeces in the pre-treatment and treatment periods respectively). Such a numerical change of 0.5 Log10 bifidobacteria/g fresh faeces is consistent with those changes observed for inulin-type fructans, which are recognised prebiotics. A possible prebiotic effect of SCF was therefore demonstrated by its stimulation of bifidobacteria numbers in the overall gut microbiota during a short-term intervention.

## Introduction

The colon contains the most abundant and diverse assemblage of bacteria in the human body [1]. Symbiotic interactions within this complex community are now recognized as important mediators of health by the scientific community [2]. Like other environmental microbial communities, the human microbiome is a complex and dynamic system that plays an important role in many aspects of host physiology [3–6]. Together with the gut immune system, colonic and mucosal microbiota contributes to a barrier that prevents pathogenic bacteria from invading the gastrointestinal (GI) tract. Generally, bacteria that have an almost exclusive saccharolytic metabolism can be generally considered as beneficial, e.g. bifidobacteria [7–11]. The intestinal microbiota salvages energy through fermentation of carbohydrates and other substrates not digested in the upper gut [12–14]. In addition, some dietary protein may reach the colon, while endogenous secretions, including mucin, provide diet-independent substrates. Many secondary plant metabolites ingested with the diet, such as polyphenolic substances, may also reach the large intestine and are subject to bacterial transformation. [14]. Colonic bacteria use a range of carbohydrate hydrolyzing enzymes to produce gases and organic acids. Dietary components that stimulate fermentation lead to an increase in bacterial mass and consequently faecal mass and, thus have a stool bulking effect [15]. It is estimated that about 30 g of bacteria are produced for every 100 g of carbohydrate that is fermented. According to Gibson and Roberfroid [16] predominant bacterial groups of the human gut microbiota may be classified into those that are either beneficial with health promoting effects; harmful or pathogenic; or those that demonstrate both or no effects.

Members of nine bacterial phyla were found to inhabit the human gastrointestinal tract, of which *Firmicutes* and *Bacteroidetes* are the dominant ones, while Actinobacteria and Proteobateria subdominants [4]. Besides the low level of biodiversity at the phylum level, the human intestinal microbiota is tremendously diverse at species and subspecies level [3]. It is characterized by a considerable interpersonal variation in bacterial species and strains [7] and, up to now, 1000 different bacterial species-level phylogenetic types have been identified in the human intestinal microbiota. However, because of a significant degree of functional redundancy, the differences in the microorganism lineage between individuals are greater than differences in the representation of the gene networks embedded in the individual microbiomes. To date, it seems clear that the relative composition of microbes within the gut microbiota is largely reliant upon food consumed, one opportunity for modulate the microbiome is through dietary change [17–20]. For this reason, dietary management of the human gut microbiota towards a more beneficial composition is an approach that can improve host health [21–22]. Bacteria such as bifidobacteria and lactobacilli have demonstrable beneficial effects through improving resistance to gut infections by inhibiting the growth of harmful bacteria [23], protecting against bacterial invasion [24], reducing cholesterol levels [25–26], improving the immune response [27] and producing vitamins within the gut [28].

The scientific understanding of dietary modulation of the human gut microbiota has been developed over several decades, with probiotics as a principal focus [18, 22]. While the role of probiotics has been widely advocated, it is possible that a greater number of food products will further exploit the prebiotic approach over the next few years. Prebiotics can also be added to many foods including those which are cooked or baked, which are more problematic for probiotic enrichment [29–30].

A maize-based soluble fibre, Soluble Corn Fibre (SCF), has been shown to be a versatile source of dietary fibre with potential selective fermentation properties as determined in vitro [31] and in vivo [32]. SCF is a glucose polymer obtained from a partially hydrolyzed starch-made glucose syrup. Possessing a high digestive tolerance after ingestion [33–35], SCF has also been shown to elicit a low post-prandial blood glucose and insulin response (i.e. reduced glycemic potential) [33]. Recently, SCF has been shown to improve bone properties [36], ameliorate the immune response in experimental colitis [35], and improve laxation [37–38], which is one of the most well noted benefits of dietary fibres. Key to the establishment of novel prebiotic products is assessment of an optimum dose level required to elicit changes in the gut microbiota during feeding. Therefore, the aim of the present pilot human study was to determine the effect of different doses of SCF for prebiotic potential in healthy volunteers and their impact on the human intestinal microbial ecosystem. Stool samples were collected before and after treatment and changes within the gut microbiota profile were determined. Nine 16S rRNA-based fluorescence in situ hybridization probes were used to target predominant groups/species of human faecal microbiota. Gastrointestinal symptoms and stool characteristics such as stool frequency, consistency, abdominal pain, intestinal bloating and flatulence were also recorded in order to assess prebiotic tolerance.

## Materials and Methods

### Selection and characteristics of study population

Twenty-four healthy volunteers (gender: 12 female, 12 male; age: mean 33, range 18 to 50 yr; body mass index (BMI): mean 23.6 kg/m^2^, range 20.1 to 29.7 kg/m^2^) were recruited from the local community. The study was approved by the University of Reading Research Ethics Committee prior to the start of the study and written informed consent was obtained from all subjects prior to commencement of selection. Inclusion criteria were males and females from 18 to 50 years of age and BMI 18.5 to <30 kg/m^2^. General health status was assessed using a pre-study medical questionnaire and putative volunteers were excluded if they fulfilled any of the following exclusion criterion: diagnosed as suffering from any chronic gastrointestinal complaints (including chronic constipation, diarrhoea or irritable bowel syndrome), diabetes or anaemia; requirement to take long-term medication active on the gastro-intestinal tract, for the treatment of cardio-vascular disease, or any other long-term medication; high blood cholesterol or use of cholesterol lowering drugs/functional foods; history of drug or alcohol misuse or alcohol consumption exceeding 14 and 21 units/week for females and males respectively; those suffering with any allergies to medication or food; smokers, and those on weight reducing diets. Females were excluded who were either planning pregnancy within six months of the start of the study, lactating, or had given birth within the preceding six months. Specified timed exclusion criteria included use of antibiotics with the previous six months, participation in any probiotic, prebiotic or laxative study in the previous three months, or intake of an experimental drug within four weeks of the study start. Consumption of any probiotic or prebiotic products and use of medication active on the gastrointestinal tract or use for the treatment of abdominal pain, intestinal discomfort, or constipation were prohibited during the four weeks prior to commencement of the study and for the duration of the study.

### Study design and treatments

**Fig 1:**
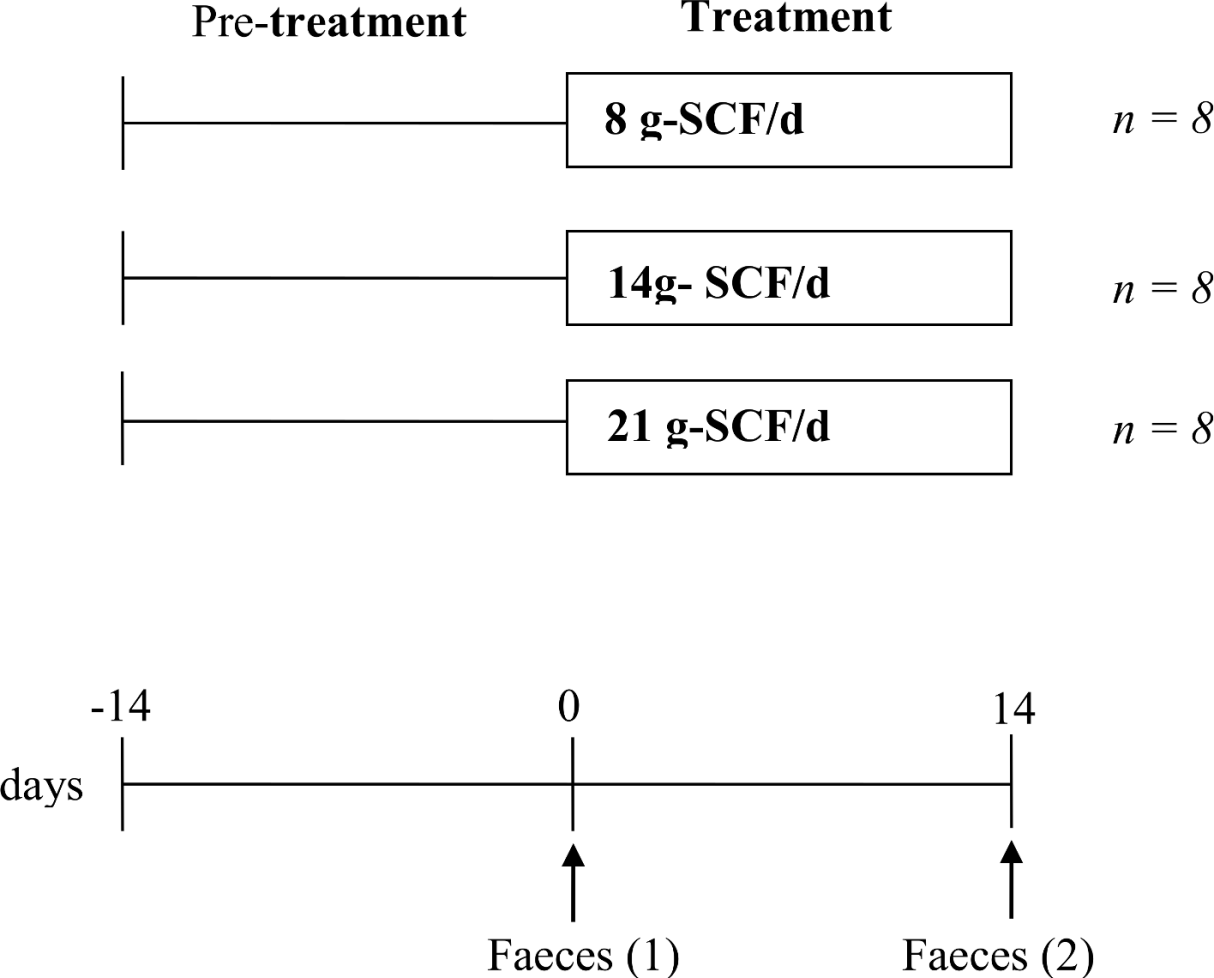
Study design of a double-blind, randomised, parallel study with eight volunteers per treatment group, and consisting of a pre-treatment and treatment period (14-days). Treatments with were 8, 14 and 21 g-SCF/d designed to 6, 12 and 18 g fibre/delivered/day from Promitor™ Soluble Corn Fibre (SCF).

The overall study design is outlined in Fig 1 and was completed in a double-blind manner, with two experimental periods (pre-treatment and treatment period) each lasting 14 days. Eight volunteers were randomly allocated on the basis of age and BMI to each of three treatment groups. The treatments were designed to provide a total of 8, 14 or 21 g from SCF (Promitor™ Soluble Corn Fibre, SCF) (equivalent to 6, 12 and 18 g/fibre delivered, respectively). Supplements were provided by Tate & Lyle, France, as dry powders and were consumed as 250 ml beverages twice daily. Volunteers were requested to substitute 500 ml of their normal daily fluid intake to compensate for the volume of water consumed with the treatments. Freshly voided faecal samples were provided by each volunteer at the end of the pre-treatment period (baseline sample) and then after 14 days of dietary intervention during the treatment period.

Faecal samples were collected in sterile plastic containers, stored in an anaerobic cabinet (10% H2; 10% CO2; 80% N2) and then processed within two hours of collection.

Throughout the pre-treatment and treatment periods, volunteers recorded details of bowel habits including stool frequency and consistency (Bristol stool scale) [39], stomach or intestinal bloating, abdominal pain, incidence and frequency of flatulence and stomach noises. This information was recorded as being indicative of digestive tolerance. In addition, volunteers recorded details of dietary intake on four days within each period (data not reported).

### Faecal preparation and enumeration of faecal microbial populations by fluorescence *in situ* hybridisation

Freshly voided faecal samples (~ 2–5 g) were diluted 1 in 10 (w/w) with sterile, anaerobic phososphate buffered saline (PBS, 0.1M; pH 7.0) and homogenised using a Stomacher 400^®^ (Seward, Norfolk, UK) for 2 min at normal speed.

Fluorescence *in situ* hybridisation (FISH) was performed as described by Costabile et al. [40]. The composition of hybridisation and wash buffers depends on the rRNA-targeted oligonucleotide probe as reported in probeBase (http://www.microbial-ecoLogy.net/probebase) and was used accordingly. All oligonucleotide probes used in the study are reported in Table 1 [40]. Briefly, aliquots (375 μl) of 1 in 10 w/w stool sample suspension in PBS, 0.1M were fixed overnight at 4°C with 4% (w/v) filtered paraformaldehyde (pH 7.2) in a ratio of 1:4 (v/v), washed twice with filtered PBS (0.2 μm pore size), resuspended in 300 ml of a PBS/ethanol mixture (1:1, v/v) and then stored at– 20°C for up to 3 months. For the hybridizations, 20 μl of each sample was pipetted onto Teflon and poly-L-lysine-coated, six-well (10 mm diameter each) slides (Tekdon Inc., Myakka City, FL). To permeabilize the cells for use with probe Lab158, samples were treated with 50 μl of lysozyme (1 mg/ml in 100 mM Tris-HCl, pH 8.0) at 37°C for 15 min before being washed (2–3 s) in water and were finally dehydrated in the ethanol series (50%, 80% and 96% v/v ethanol, 3 min each). A probe/hybridization buffer mixture (5 ng/5 μl of a 50 ng/μl probe in stock solution plus 45 μl of hybridization buffer) was applied onto the surface of each well. Hybridizations were performed for 4 h in an ISO20 oven (Grant Boekel). For the washing step, slides were placed in 50 ml of wash buffer containing 20 μl of 4, 6-diamidino-2-phenylindole di-hydrochloride (DAPI; 50 ng/μl; Sigma, UK) for 15 min. They were then washed (2–3 s) in ice-cold water and dried under a stream of compressed air. Five μl of antifade reagent (polyvinyl alcohol mounting medium with DABCOTM antifading, Sigma) was added to each well and a coverslip was applied. Slides were stored in the dark at 4°C (for a maximum of 3 days) until cells were counted under a Nikon E400 Eclipse microscope. DAPI slides were visualized with the aid of a DM 400 filter and probe slides with the aid of a DM 575 filter.

**Table 1:** Probes used for Fluorescence *in* situ hybridization (FISH) analysis of bacterial populations in human faeces.

Short name	Accession no.*	Full name	Target species	Sequence (5´ to 3´)
**Bif164**	**pB-00037**	**S-G-Bif-0164-a-A-18**	**Most *Bifidobacterium* spp. and *Parascardovia denticolens***	**CATCCGGCATTACCACCC**
**Lab158**	**ND**	**S-G-Lab-0158-a-A-20**	**Most *Lactobacillus*, *Leuconostoc* and *Weissella* spp.; *Lactococcus lactis*; all *Vagococcus*, *Enterococcus*, *Melisococcus*, *Tetragenococcus*, *Catellicoccus*, *Pediococcus* and *Paralactobacillus* spp.**	**GGTATTAGCAYCTGTTTCCA**
**Bac303**	**pB-00031**	S-*-Bacto-0303-a-A-17	**Most *Bacteroides sensu stricto* and *Prevotella* spp.; all *Parabacteroides*; *Barnesiella viscericola* and *Odoribacter splanchnicus***	**CCAATGTGGGGGACCTT**
**Chis150**	**pB-00962**	S-*-Chis-0150-a-A-23	**Most members of *Clostridium* cluster I; all members of *Clostridium* cluster II; *Clostridium tyrobutyricum*; *Adhaeribacter aquaticus* and *Flexibacter canadensis* (family *Flexibacteriaceae*); [*Eubacterium*] *combesii* (family *Propionibacteriaceae*)**	**TTATGCGGTATTAATCTYCCTTT**
**Ato291**	**pB-00943**	S-*-Ato-0291-a-A-17	***Atopobium*, *Colinsella*, *Olsenella* and *Eggerthella* spp.; *Cryptobacterium curtum*; *Mycoplasma equigenitalium* and *Mycoplasma elephantis***	**GGTCGGTCTCTCAACCC**
**Erec482**	**pB-00963**	S-*-Erec-0482-a-A-19	**Most members of *Clostridium* cluster XIVa; *Syntrophococcus sucromutans*, [*Bacteroides*] *galacturonicus* and [*Bacteroides*] *xylanolyticus*, *Lachnospira pectinschiza* and *Clostridium saccharolyticum***	**GCTTCTTAGTCARGTACCG**
**Eub I-II-III**	**pB-00159-160-161**	***S-D-Bact-0338-a-A-18***	***Most domain bacteria***	**GCT GCC TCC CGT AGG AGT**
**Rrec584**	**ND**	**ND**	***Roseburia* genus**	**TCAGACTTGCCG(C/T)ACCGC**
**EC1531**	**ND**	**L-S-Eco-1531-a-A-21**	***Escherichia coli***	**CACCGTAGTGCCTCGTCATC**

*ND, No information relating to these probes has been deposited in probeBase (http://www.microbial-ecology.net/probebase).

‡These probes were used together in equimolar concentrations (both at 50 ng μl^−1^).

Formamide (20%) was included in the hybridization buffer.

The probes were commercially synthesized and labelled at the 5’ end with the fluorescent dye Cy3 (Sigma-Aldrich, Poole, Dorset, UK).

### Faecal moisture content

Faecal moisture content was determined following oven drying a representative sub-sample of each faecal sample in a fan-assisted oven at 100°C for 18 h.

### Data transformations and statistical analysis

Data for the enumeration of the key beneficial groups of faecal bacteria were expressed as Log10 cells/g fresh faeces. Within each treatment group the results for digestive tolerance and faecal bacteriology were analysed using paired t-tests (i.e. pre-treatment vs. treatment values) within the Minitab^®^ Software (Windows V15). Significance was set at P<0.05.

## Results

### Bowel habit and indicative digestive tolerance

A summary of the mean bowel habit and indicative digestive tolerance recorded within each treatment group (i.e. 8 volunteers/treatment group) in both the 14-day pre-treatment and treatment periods is shown in Table 2. With the exception of bowel movement recordings (number/day) and stool consistency (score 1 to 7 representing hard to entirely liquid stools), volunteer responses were ranked to give rise to scores, whereby 0 indicated no symptoms, 1 indicated very mild symptoms and 10 indicated very severe symptoms. The data show that there was no significant effect (P>0.05) of treatment on the mean number of bowel movements per day (1.34, 1.53, and 1.47 no/day, across respective treatments) or on stool consistency (3.7, 3.8, and 4.1, across respective treatments), values of which were similar across all treatment groups (overall mean across all treatment groups in the pre-treatment and treatment periods respectively). Determination of stool dry weight also showed that there was no significant effect (P>0.05) of treatment on this between treatment groups (23.7, 28.3, and 27.1 g/100g, respectively) and that values were similar across all treatments (overall mean across all treatments 26.3, 25.6 and 26.2 g/100g fresh stool in the pre-treatment and treatment periods respectively). There was a significant effect on flatulence with the 14 g-SCF treatment, 1.33 and 2.28 in the pre-treatment and treatment periods). However, the effect of the treatment on flatulence was small, with overall values reported as none to very mild in the 14g-SCF treatment, and furthermore was not statistically significant in the 21g-SCF treatment. There was no significant effect of treatment on bloating, abdominal pain or stomach occurrences in any treatment group.

**Table 2:** Summary of the mean stool frequency and consistency, and indicative digestive tolerance recorded daily by eight volunteers per treatment group for each of 14 days in the pre-treatment (baseline) and treatment periods. Values in parenthesis are standard error (SE) values.

	8 g-SCF (6g/fibre/delivered/d)		14 g-SCF (12g/fibre/delivered/d)		21 g-SCF (18g/fibre/delivered/d)	
Parameter	Pre-treatment	Treatment	Pre-treatment	Treatment	Pre-treatment	Treatment
Bowel movements (no./day)	1.34 (0.13)	1.34 (0.14)	1.43 (0.16)	1.53 (0.15)	1.35 (0.21)	1.47 (0.16)
Stool consistency^1^	3.6 (0.20)	3.7 (0.20)	3.7 (0.26)	3.8 (0.15)	3.8 (0.22)	4.1 (0.22)
Bloating^2^	0.51 (0.27)	0.89 (0.60)	0.12 (0.11)	0.42 (0.17)	1.15 (0.52)	0.93 (0.31)
Abdominal pain^2^	0.77 (0.46)	0.61 (0.46)	0.05 (0.04)	0.23 (0.16)	0.98 (0.47)	0.71 (0.28)
Flatulence^2^	1.54 (0.41)	1.84 (0.66)	1.33 (0.36)	2.28 (0.38)**	1.46 (0.31)	2.50 (0.78)
Stomach noises^2^	0.73 (0.25)	0.92 (0.45)	0.40 (0.15)	0.96 (0.37)	0.49 (0.20)	1.30 (0.67)
Stool dry weight (g/100 g)^3^	26.3 (2.71)	23.7 (1.98)	25.6 (3.37)	28.3 (2.08)	26.2 (2.00)	27.1 (1.63)

Promitor™ Soluble Corn Fibre (SCF) 8, 14 and 21 g-SCF, treatments designed to provide 6, 12 and 18 g fibre/delivered/day from SCF.

^1^, estimated using the Bristol stool chart (scale with seven stool types; Type 1 –hard to Type 7 –entirely liquid).

^2^, overall scale from 0 to 10 (0—no symptoms; 1 –very mild symptoms to 10 –very severe symptoms).

^3^,stool dry weight determined following oven drying at 100°C for 18 h.

*, P<0.05.

**, P<0.01 (vs pre-treatment).

### Enumeration of faecal microbial populations

Effects of the three treatments studied on predominant groups of the human gut microbiota are summarised in Table 3 and expressed as Log_10_ cells/g fresh faeces. FISH analysis showed that there was a significant increase (P<0.05) in concentration of health-promoting genus *Bifidobacterium* (Bif164) following consumption of the 6 g fibre/delivered/day when compared to the pre-treatment period. However, there was no significant increase (P>0.05) in numbers of bifidobacteria for any of the other treatment groups, although the 18 g/ fibre/delivered/day indicated a trend (P = 0.083) for an increase (mean 9.41 and 9.65 Log_10_ cells/g fresh faeces in the pre-treatment and treatment periods respectively). There was no significant effect (P>0.05) on lactobacilli although there was a trend (P = 0.09) for values to increase following 14-d consumption of the 6 g/ fibre/delivered/day (mean 8.07 and 8.37 Log_10_ cells/g fresh faeces in the pre-treatment and treatment periods respectively). A significant decrease (P<0.05) in numbers of *C*. *histolyticum /perfringens* group following consumption of the 12 g/ fibre/delivered/day was observed (mean 7.72 and 7.19 Log_10_ cells/g fresh faeces in the pre-treatment and treatment periods respectively. *Eubacterium rectale-Clostridium coccoides group* enumerated by the Erec482 probe did not significantly influence (P > 0.05) their concentrations. A similar behaviour was demonstrated for *Bacteroides-Prevotella* group (detected by Bac303), Rrec584 probe enumerating the butyrate-producing *E*. *rectale-Roseburia* group, also a component of cluster XIVa, the *Atopobium* group (detected by Ato291) comprises bacteria of the *Coriobacteriaceae* family, belonging to *Coriobacterium*, *Atopobium* and *Collinsella gen*era and *E*. *coli* (detected by Ecoli1351), whose concentrations were stably maintained during the study.

**Table 3:** Enumeration of faecal microbial populations in fresh stool samples collected from eight volunteers per treatment group both before (pre-treatment/baseline) and following 14-day dietary intervention (treatment) in a double-blind, randomised, parallel study. Stool bacterial numbers as determined by fluorescence *in situ* hybridisation are expressed as mean Log_10_ cells/g fresh faeces (values in parenthesis are standard error (SE) values).

Bacterial group	Probe	8 g-SCF		14 g-SCF		21 g-SCF	
		Pre-treatment	Treatment†	Pre-treatment	Treatment	Pre-treatment	Treatment
Total bacteria	Eub338 I-II-III	11.0 (0.07)	11.0 (0.09)	11.1 (0.08)	11.1 (0.12)	11.1 (0.10)	11.2 (0.06)
*Bacteroides*/*Provotella* gp.	Bac303	10.1 (0.05)	10.2 (0.07)	10.4 (0.11)	10.2 (0.06)	10.3 (0.07)	10.4 (0.08)
Lactobacilli/enterococci	Lab158	8.07 (0.20)	8.37 (0.17)	7.87 (0.23)	7.71 (0.33)	8.08 (0.12)	8.07 (0.20)
*Bifidobacterium* spp.	Bif164	9.25 (0.27)	9.73 (0.22)*	9.26 (0.26)	9.35 (0.43)	9.41 (0.11)	9.65 (0.09)
*Coriobacteriaceae*	Ato291	9.43 (0.10)	9.37 (0.08)	9.47 (0.16)	9.39 (0.17)	9.46 (0.12)	9.52 (0.08)
*C*. *histolyticum /perfringens* gp.	Chis150	7.34 (0.15)	7.22 (0.14)	7.72 (0.16)	7.19 (0.22)*	7.51 (0.33)	7.59 (0.31)
*Clostridium coccoides-Eubacterium rectale* gp.	Erec482	10.3 (0.09)	10.3 (0.08)	10.4 (0.06)	10.4 (0.07)	10.4 (0.07)	10.3 (0.08)
*Eubacterium rectale-Roseburia* sub-gp.	Rrec584	9.87 (0.19)	9.97 (0.11)	10.0 (0.08)	9.95 (0.12)	10.0 (0.08)	9.94 (0.10)
*Escherichia coli*	Ecoli1351	8.05 (0.28)	8.00 (0.23)	7.68 (0.16)	7.68 (0.20)	10.0 (0.08)	9.94 (0.10)

**†**Treatments with 8, 14 and 21 g-SCF designed to provide 6, 12 and 18 g fibre/delivered/day from Promitor™ Soluble Corn Fibre (SCF).

*, P<0.05 (vs pre-treatment).

## Discussion

### Bowel habits and indicative digestive tolerance

The results of this ‘pilot’ study has demonstrated that SCF was well tolerated at daily dose levels of 6, 12 and 18 g/fibre/delivered/d following 14-day dietary interventions in healthy adults. Overall volunteer responses for stomach/intestinal bloating, abdominal pain, flatulence and stomach noises were recorded as none to mild (i.e. overall score <4). These results are in agreement with previous studies [32, 33, 41, 42, 43, 44] on the digestive tolerance of prebiotics, including those determined in a single amount up to 65 g fibre/d [45]. The authors also concluded that SCF was well tolerated at daily amounts as high as 65g [45], whereas some candidate prebiotic fibres have been shown to induce a mild laxation effect, increasing stool frequency and weight when consumed at high doses (>20 g/day)

In addition to the high digestive tolerance afforded by SCF, the present study showed that treatments were well tolerated as seen by no significant adverse effect on bowel habits, as determined by stool frequency and consistency, with similar values recorded both before and following 14-days of intervention. The values of 1.41 and 1.46 bowel movements per day recorded across all treatments in the pre-treatment and treatment periods are within the range previously reported [12–13]. For adults across Western countries, these authors state that the modal stool frequency is 1 per day; for the population as a whole the range is 3 stools per day to 3 stools per week [12–13]. Stool consistency was unchanged by dietary intervention and it is interesting to note that there was close agreement in the findings of the subjective assessment of stool consistency using the Bristol stool chart (recorded daily across 14 days during the pre-treatment and treatment periods) and objective assessment of stool moisture content (i.e. oven drying at 100°C for 18 h). Determination of stool consistency is an important attribute in the overall assessment of digestive tolerance and is related to stool moisture content (normal range, 70–80 g moisture/100 g fresh stool [13]). Therefore, the present study validates the approach of recording a subjective assessment of stool consistency in bowel habit diaries for use in dietary intervention studies since it is impracticable to routinely oven dry stool samples.

### Assessment of prebiotic potential

Prebiotics are defined as ‘non digestible food ingredients that are selectively metabolised by colonic bacteria that have the capacity to improve health’ [16,17]. Prebiotics, in part due to their function as a special type of soluble fibre, can contribute to the health of the general population; and a number of challenges must be addressed in order to fully realize prebiotic benefits, including the need for greater awareness of the accumulated evidence on prebiotics among policy makers [22]. According to Tuohy et al. [42], the most frequently tested prebiotics are the non-digestible oligosaccharides, such as the fructans and galactans, which confer selective increases in bifidobacteria following short feeding periods.

While many non-digestible ‘by-pass’ carbohydrate materials/products may be potential candidate prebiotics, this classification cannot be extended to all non-digestible carbohydrates and dietary fibres [16]. Key to the establishment of novel prebiotic products is assessment of an optimum dose level required to elicit changes away from a gut flora dominated by potentially harmful bacteria towards a more benign, or beneficial, composition, since there is currently no recommended daily intake of prebiotics [16]. The current study examined the effect of 3 doses of SCF (6g, 12g and 18 g fibre/delivered/day from SCF) on the human gut microbiota following a 14-d intervention. In the present study, fluorescence *in situ* hybridization (FISH) method instead of 16S sequencing was used to better focus on some key taxa of interest and to obtain numerical counts of bacterial groups that are considered relevant based on previous work. Furthermore, 16S sequencing approaches might lead to bias and has previously led to under-representation of bifidobacteria. Numbers of bifidobacteria were shown to be significantly higher for the 8 g-SCF treatment. The numerical increase of 0.5 Log_10_ bifidobacteria/g fresh stool is consistent with numerical changes of 0.5–1.0 Log_10_ observed for inulin-type fructans [33] which are thought to exert their prebiotic effects at doses higher than 5 g/d–usually 8 g/d. According to Kolida and Gibson [46] such a change in numbers of bifidobacteria ‘constitutes a major shift in the gut microbiota towards a “healthier” composition’. This prebiotic effect concurs with that demonstrated *in vitro*, whereby relative numbers of bifidobacteria were shown to increase [46]. In addition, results of *in vivo* and *in vitro* studies show that SCF promotes the production of SCFA including butyrate, which are positively associated with colonic health. However, increasing levels of SCFA was generally not associated with further significant changes in numbers of bifidobacteria [47].

This is concordant with other in vivo intervention studies with fructans which often reported increases between 0.5–1.0 Log_10_ bifidobacteria counts [46]. However, the magnitude of increase seems to depend on the baseline values and higher increases are often observed in volunteers with low initial bifidobacteria numbers. While the bifidogenic potential is well-proven the effect of consumption of SCF on other bacteria groups is less well established. A decrease in clostridia was mainly reported at the dose of 12 g/fibre/delivered/day compared to the pre-treatment. However, it cannot be determined whether the decrease in clostridia after SCF intake should be seen as detrimental.

The present study has demonstrated that the optimum prebiotic dose-response was 6 g fibre/delivered/day from SCF. A low dose of SCF was shown to stimulate bifidobacteria in the gut microbiota. However, such an increase in numbers of bifidobacteria has been demonstrated over a wide range of doses in previous prebiotic studies [47–48]. The range in dose levels has been estimated to be 3–20 g/d [33] and 4–40 g/d [34] for inulin, although for individual prebiotics, differences in optimal dose level are likely to vary according to several factors including, degree of polymerisation and molecular weight [42]. An increase in the dose of SCF greater than 6 g fibre/delivered/day was generally not associated with further stimulation of the bacterial groups included for study here. This absence of a quantifiable or defined effect with increasing prebiotic inclusion is in general agreement with previously published studies investigating prebiotic dose-response effects [49–51]. In a placebo-controlled, cross-over study (n = 30), Kolida et al. [46] showed that there was no dose-response relationship following ingestion of 8 g inulin/d. Comparing daily doses of 5, 10, 20 and 40 g gum Arabic/d [52] the authors showed that the optimum daily dose was approximately 10 g/d. The explanation for the general absence of dose-response relationships is not widely understood. One such explanation proposed by Calame et al. [52] is at high doses other bacterial strains have easier access to the substrate and subsequently, less becomes available for the ones determined within their study. This is a possibility requiring further research.

In the current study numbers of faecal lactobacilli/enterococci were unchanged compared to pre-treatment values although a mean numerical increase of 0.3 Log_10_ cells/g fresh stool was recorded for the 6 g/fibre/delivered/day as compared to the pre-treatment. Since no significant effect of dietary intervention was observed on numbers of total bacteria results of the present study suggest that consumption of 6 g/ fibre/delivered/day from SCF is selectively fermented by *Bifidobacterium* spp. and that their relative contribution to the overall gut microbiota is thereby increased.

In conclusion, SCF is a fibre source, which was well tolerated in doses as high as 18 g/ fibre/delivered/day in this study. A possible prebiotic effect was demonstrated by its stimulation of numbers of bifidobacteria in the overall gut microbiota following consumption of 6 g fibre/delivered/day. Further work is required to demonstrate the effect of SCF on other bacterial populations and in a placebo-controlled, cross over study–to assess specificity and therefore to support the potential application of this novel fiber as prebiotic in human nutrition.
